# Who gets the last bed in the intensive care unit: Nonmedical factors in delaying an operation

**DOI:** 10.1016/j.xjon.2025.06.008

**Published:** 2025-06-20

**Authors:** Brandi B. Scully, Joshua B. Goldberg, Robert M. Sade

**Affiliations:** a Department of Surgery, Johns Hopkins University, St Petersburg, Fla; b Department of Cardiothoracic Surgery, Weill-Cornell Medicine, New York Presbyterian Hospital, New York, NY; c Division of Cardiothoracic Surgery, Department of Surgery, Medical University of South Carolina, Charleston, SC

**Keywords:** ethics, intensive care decision making, professionalism

**Figure:**
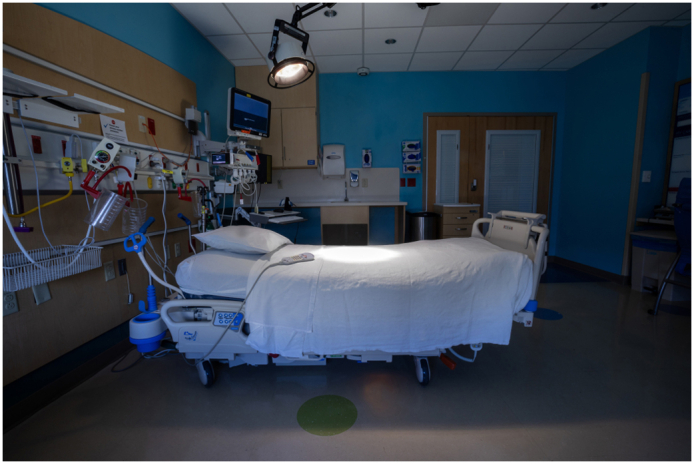
Only 1 bed is available in the ICU: Who should get it?

**Central Message**

When only 1 bed is available in the ICU, more than 1 patient needs it, and clinical situations are nearly equivalent, nonmedical considerations come into play.

Robert M. Sade, MD

One of the most common problems in cardiothoracic surgery is how to allocate one of a hospital's most scarce resources: intensive care unit (ICU) beds. When the unit's capacity is down to the last bed, what are the appropriate criteria for determining which of 2 or more patients in need of critical care should be assigned that bed? Many actors play a role in making that determination, but ultimately, the decision will be made by a Figure 1, usually the medical director of the unit. When faced with the sudden reduction of 2 available beds to 1 and 2 similarly situated preoperative patients who both require an ICU bed, what criteria should the responsible decision maker use to give the bed to 1 patient and deny it to the other? The vignette below is typical of the difficult decisions that occur frequently in most ICUs.

**Figure 1:**
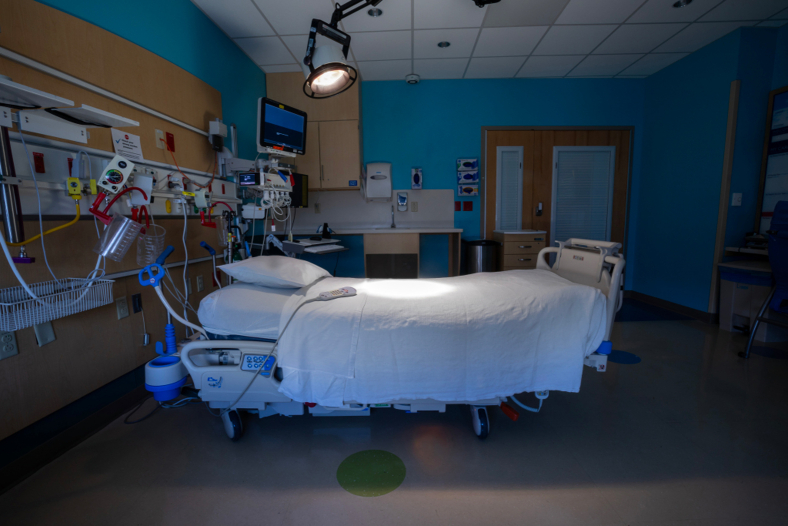
Only 1 bed is available in the ICU: Who should get it?

## Case

Dr Valerie Goodwill is the head anesthesiologist, overseeing the operating room (OR) schedule today, and serves as the medical director of the surgical ICU. The schedule is full, and when she arrives at 6 am, Dr Goodwill is confronted with a common problem: There are not enough ICU beds to do all the cases. There had been 2 beds for today's thoracic surgery cases until 4:30 am, when a new trauma patient was rushed to the OR and will need 1 of the beds, leaving only 1 bed for the 2 “elective” cases. Dr Goodwill has to decide between Dr Breatheright's pneumonectomy patient and Dr Swallow's open Ivor-Lewis esophagogastrectomy patient, both of whom have cancer requiring an operation soon, and both have a good chance for curative resection. Dr Goodwill explores potential avenues for generating another bed space, including looking for lower-acuity patients to transfer out of the unit, but none is even close to eligibility for transfer. For reasons Dr Goodwill does not understand, the 2 attendings have talked and could not reach an agreement about which patient's procedure should be postponed. She talks with both of them about postponing their patient for a few days until a bed becomes available, but neither is willing to relinquish their claim on the remaining bed.

Dr Breatheright's patient, Lucille Webster, is the 70-year-old mother of a thoracic surgeon from another state, who cleared his patient schedule for a week and flew into town to be with his mother for her surgery. She has been a heavy smoker for many years, but stopped when she developed a severe cough that led to a pulmonary workup. She was found to be frail and to have locally advanced left hilar adenocarcinoma and was treated with neoadjuvent chemo-immunotherapy, which was completed 5 weeks ago; treatment protocol requires operation within 6 weeks of completing therapy. She will require a stay in the ICU because results of pulmonary function tests and a V/Q scan make her a high risk patient for a pneumonectomy. Allowing her surgery to proceed would not only be a courtesy to another physician, but to all of his patients who would be affected by rescheduling.

Dr Swallow's patient, Luis Suarez, is a 55-year-old naturalized citizen with marginal English language skills. He has never gotten a fair deal. He had epigastric pain for more than a year, but had a hard time getting clinic appointments because his job did not provide paid sick leave and he had no insurance. When he finally saw a doctor, he received only a cursory examination and a prescription for ulcer medication. Eventually, substantial weight loss led to endoscopy that found locally advanced esophageal cancer. He was treated with neoadjuvant chemo-radiotherapy, completed 6 weeks ago; treatment protocol suggests operation within 6 weeks of completing therapy, after which the risk of postoperative complications increases. He requires a stay in the ICU because his preoperative cardiac evaluation showed cardiomegaly and a left ventricular ejection fraction of 35%, so he is regarded as a high-risk patient for the proposed esophagogastrectomy. It is not fair to cancel him now; he has been through too much already.

All the other special care units, for example, the postanesthesia care unit and the emergency department, are full, so no backup care area is available. Dr Goodwill is leaning toward canceling Dr Swallow's operation in favor of Dr Breatheright's. Is that the right decision?

PRO

Brandi B. Scully, MD, MS

Dr Goodwill should give the ICU bed to Dr Breatheright's patient, Lucille Webster.

Both patients have similar prognoses and need for an operation. Family support will be critical in the postoperative period. Ms Webster's surgery proceeding first leads to the greatest overall benefit for the greatest number of people, and it supports her autonomy as well as psychological well-being.

One of the many guidelines for ICU bed allocation published during COVID-19, the ANZICS guidelines, states that “clinical prioritization should be the initial approach to determine access to intensive care when resources are limited.”^1^ Yet these 2 patients’ medical situations are essentially equivalent. Nevertheless, Ms Webster should have priority.

Her son, the thoracic surgeon, should be available to provide critical support. A growing knowledge of the importance of family member presence on clinical outcomes has even led to an emphasis on hospital room design to allow for enhanced family involvement.^2^ We learned during the COVID-19 pandemic that restricting family member access to ICU patients can have devastating consequences. It impacts patients’ emotional and mental health, hinders communication and the ability of family members to advocate for their loved ones to the health care team, impacts safety and quality of care, and erodes trust in the health care system.^3^ Although Mr Suarez will also benefit from family support, we have no indication from the information provided that his family would not be equally able to support him this week versus next.

Moreover, for Ms Webster, her son is not only her closest family member, he is a subject matter expert. Who better to help shepherd Ms Webster through a potentially treacherous postpneumonectomy course? He draws from a deep well of knowledge

The issue of whether Ms Webster's son, as a thoracic surgeon, deserves priority because he has cleared his schedule for the week is another consideration. Delaying Ms Webster's case would directly impact her son's patients, who may be canceled themselves if he needs to rearrange his schedule again. The domino effect of downstream patient delays makes the consideration of canceling Ms Webster even less appealing.

However, should Ms Webster's son's patients be a consideration at all? Although a utilitarian approach seeks the greatest benefit for the most patients, extending this appeal to patients in a different hospital takes the focus off the 2 patients in front of us. It is very likely that Ms Webster's son, as well as his patients, will be negatively affected by her surgery being postponed, and we do not know that the same domino effect would take place in the case of postponing Mr Suarez's operation.

In contrast, a deontological appeal to moral obligations compels us to provide care for Mr Suarez, who lacks the same means as Ms Webster and who has already suffered within the health care system from having his concerns dismissed and his treatment delayed until his cancer was locally advanced. Moral injury to the physician might occur here from layering harm on top of harm.

It is important to remember that cancellation of major surgery for administrative reasons is not just an inconvenience. It causes well-documented patient harm. Ivarsson and colleagues^4^ have reported patients scheduled for heart surgery react negatively on cancellation with anxiety, disappointment, and fear, especially if the reason for cancellation was organizational in origin. Of particular note, when women's operations were cancelled, they had a much higher degree of depression compared with other patients.^5^ Ms Webster, as an elderly woman, would sustain greater harm than Mr Suarez from even a small delay.

Cancellation of major surgery due to lack of beds also can lead to inferior clinical results, as Magnusson and colleagues^5^ demonstrated. They asked the question, “Will a psychologically less than optimally prepared patient experience more complications and inferior final results?” and found significantly more complications and also a strong tendency toward inferior long-term well-being. They note that, “at the time of scheduling, an elective patient is given a precise point in time on which to focus all her psychological stamina. She is about to accept and allow a frightful, dangerous, and potentially lethal procedure. When this surgery is canceled, often on precariously short notice, there is a tremendous letdown and disappointment.”^5^

Ms Webster's autonomy is also important to consider here. Aquino and colleagues^6^ reviewed 21 international guidelines for allocation of critical care resources during COVID-19 and found that “The principle of respect for autonomy or self-determination is the ethical concept mentioned by the largest number of guidelines,” being seen in 19 of 21 sets of guidelines reviewed.^6^ And as Magnusson and colleagues^5^ assert, “Vital interests, eg, risk of severe complications, anxiety, and psychological distress, are at stake when administrative decisions to postpone treatment are taken. Respect for autonomy in recognition of a patient's wish to be in control is also a vital ethical interest to take into consideration.”^5^ In this case, autonomy for Ms Webster includes the ability to have her support people, like her son, at her bedside.

Although Ms Webster may have more resources than Mr Suarez to reschedule her surgery, in this instance, her greater resources and access will not protect her from suffering greater harm. Ms Webster is particularly vulnerable. She is an elderly, frail female, which puts her at higher risk of depression if canceled, and she has put forth significant effort to have her son come to be her support person through this significant trial.

She also has the added stigma of lung cancer, where patients can be discriminated against due to the specious argument that they are the cause of their own suffering. She should not be penalized for this.^7^

Mr Suarez's delay in care and socioeconomic disadvantages have undoubtedly adversely affected his care thus far, but that does not change the likelihood that he will not benefit as much from this ICU bed because of his advantages over Ms Webster in terms of age and gender. His outcome will be less likely to be negatively impacted by a short delay.

Aquino and colleagues^6^ note that ICU bed allocation is not a way to combat social or economic injustice.^6^ That is the space of public health policy and preventative ethics:

“Regarding the tension between maximizing utility and justice, we argue that the consequences of structural inequities cannot be appropriately or comprehensively rectified at the point of ICU allocation…preventative interventions may include…improved health care access for socioeconomically disadvantaged groups, and dedicated support for people with disability, among others.”^6^

This is one of the reasons why Valera and colleagues^8^ advised against labeling the last bed in the ICU as a dilemma.^8^ They argue that each patient should be evaluated on their own merit.

Both of these patients need an operation, and both will get an operation. The issue we are debating is who is going to go first. Ms Webster has ample reasons to get the ICU bed. Her gender and age make her more vulnerable, both to postoperative complications and to harm from canceling her procedure. Canceling her case undermines her autonomy by compromising her control over her environment and preventing her well-laid plans for support from taking place.

The preponderance of the evidence for prioritizing Ms Webster in this case is why Dr Goodwill is leaning toward postponing Dr Swallow's operation in favor of Dr Breatheright's. An ethical analysis suggests that Ms Webster stands to gain the most clinical and psychological benefits from this ICU bed, and that it results in the greatest amount of good for the greatest number of patients, while not compromising Mr Suarez's safety or long-term outcome.

CON

Joshua Goldberg, MD

Dr Goodwill should allocate the ICU bed to Mr Suarez, allowing his case to proceed.

A nuanced aspect of this debate is based on several assumptions from the scenario. As written, one may assume that Mr Suarez and Ms Webster have starkly different backgrounds in terms of health care literacy, English proficiency, and socioeconomic resources. Mr Suarez's English is “marginal,” and, as is typically the case when health care providers do not speak the patient's primary language, reliance on the availability of English-proficient family members, Spanish-speaking health care providers, or translator services, all of which can result in inadequate communication.

Socioeconomic disadvantages have been strongly associated with compromised health care outcomes in a diverse spectrum of medical conditions, including cancer.^9^^,^^10^ Explanations for this association include decreased preventive health care and health care screening, greater challenges to adherence to treatment regimens due to difficulties in arranging and paying for appointments, and transportation, among others. The scenario clearly describes the socioeconomic challenges that Mr Suarez faced in his cancer care as indicated by difficulty scheduling appointments, getting time off work that caused delays in his diagnosis and treatment, and ultimately preparing him for surgery. Furthermore, the same socioeconomic stressors that impact Mr Suarez also likely impact his family and support network, manifested as challenges to taking time off from work to accompany Mr Suarez to his appointments, the cost of childcare for family members who accompany Mr Suarez, and the direct costs of transportation, parking, and so forth. This has created a foundation of inequity, maleficence, and injustice that are likely not present with Ms Webster.

Socioeconomic stressors and challenges with health care literacy likely contribute to compromised health care. As with socioeconomic status, there is an association with language barriers and compromised medical outcomes.^11^^,^^12^ Even with professional interpreter services, a communication gap remains between physician and patient that often compromises understanding of the medical diagnosis and treatment. According to a limited body of data, in-person professional interpreters are the most effective at accurately translating complex medical information between physician and patient when a language barrier is present.^13^^,^^14^ Unfortunately, in-person professional translator services are rarely available or used, because many providers rely on less efficacious translation modalities such as videoconferencing applications, telephone, untrained medical professionals (nurses or technicians), or a bilingual family member. Unfortunately, much detailed medical information is, quite literally, lost in translation. Thus, when dealing with a complex medical diagnosis and treatment plan, such as cancer, this communication barrier can have profound negative impact on health care delivery.

Poor health care literacy is also associated with compromised medical outcomes, especially with cancer, given the complexity of the work-up, diagnosis, and treatment.^15^ Combined with a language barrier, a large communication barrier undoubtedly exists between Mr Suarez and the health care system. The language barrier further challenges the ability to overcome marginal health care literacy in terms of providing Mr Suarez sufficient information so he can make an autonomous, informed medical decision. This barrier most certainly impacted the timeliness of his diagnosis and treatment. Thus, the foundation of inequity, injustice, and maleficence created by Mr Suarez's socioeconomic status is further exacerbated by the lack of autonomy imposed by the language barrier and inadequate health care literacy.

The “raw deal” mentioned in the scenario is the culmination of the socioeconomic stressors, language barrier, and inadequate health care literacy that have made Mr Suarez's cancer care journey challenging. Although these factors inevitably contributed to the delays in diagnosis and treatment, Mr Suarez's past challenges have no bearing on the decision of who should get the ICU bed. Regardless of past challenges, advantages, or disadvantages, both patients are in need of an ICU bed for their necessary cancer surgery. The real question is, who will be able to better tolerate a delay, both medically and socially, as well as economically? Independent of treatment protocol timing, both patients are of equivalent medical urgency. However, the medical and socioeconomic injustices sustained by Mr Suarez will be much greater if there is a delay. The language barrier and compromised health care literacy create the greatest potential for increased confusion, miscommunication, and the risk of nonadherence and future no-show for a rescheduled surgery. Ms Webster's son will undoubtedly understand the necessary delay as he has encountered similar situations in his own practice. Furthermore, the financial burden for Mr Suarez due to rescheduling will be significantly greater than for Ms Webster, including direct costs such as transportation and parking, as well as indirect costs to his family, such as rescheduling time off work, childcare, and their own parking and transportation expenses.

The facts that Ms Webster's son is a thoracic surgeon and that a delay may impact his patients are irrelevant. He is not directly involved in his mother's medical care. He should be viewed no differently than any other family member who provides social support.

## Concluding Remarks

Robert M. Sade, MD

Although not an everyday occurrence, situations like that faced by Dr Goodwill are not rare. Even for patients who are not as closely matched clinically as Ms Webster and Mr Suarez, competition for an ICU bed can lead to similar quandaries for operating room and ICU managers. When acuity differences do not dictate priority, allocation to 1 patient or another can be guided by non-medical factors such as those considered by our essayists.

In our vignette, Dr Goodwill is asked to decide which of 2 patients should be assigned the 1 remaining ICU bed, but instead of making a decision, Dr Goodwill could consider escalating such conflicts to the department chair or other senior administrator. However, she is in charge of the operating schedule and also serves as the director of the surgical ICU, which gives her the authority to make decisions about priorities for the single available bed. She has heard persuasive arguments on both sides of the debate and is undoubtedly frustrated by the surgeons' inability to reach an agreement on their own.

Setting aside the possibility of passing the decision to someone else, Dr Goodwill appears to have at least 3 other options. She could determine that one argument is more persuasive than the other (although many readers will view them as well-balanced) and allow that patient to proceed to surgery, which would, at the very least, disappoint the surgeon whose patient was not selected. Alternatively, she could inform the 2 surgeons and their teams that the bed will not be allocated to either until they agree on which patient will go first. However, this could risk extending the impasse to the point where no operation is performed, benefiting no one. Finally, she might conclude that trying to differentiate between the patients is a futile exercise because neither medical nor nonmedical grounds are decisive, implying that the clinically equivalent patients have equal claims for priority, making any choice arbitrary. Dr Goodwill's Solomonic choice might simply be to assign heads and tails to the 2 patients and flip a coin.

## Conflict of Interest Statement

The authors reported no conflicts of interest.

The *Journal* policy requires editors and reviewers to disclose conflicts of interest and to decline handling or reviewing manuscripts for which they may have a conflict of interest. The editors and reviewers of this article have no conflicts of interest.
